# Electrochemical Properties of LLTO/Fluoropolymer-Shell Cellulose-Core Fibrous Membrane for Separator of High Performance Lithium-Ion Battery

**DOI:** 10.3390/ma9020075

**Published:** 2016-01-26

**Authors:** Fenglin Huang, Wenting Liu, Peiying Li, Jinxia Ning, Qufu Wei

**Affiliations:** Key Laboratory of Eco-Textiles, Ministry of Education, Jiangnan University, Wuxi 214122, China; flhuang@jiangnan.edu.cn (F.H.); 6140703005@vip.jiangnan.edu.cn (W.L.); 6130703015@vip.jiangnan.edu.cn (P.L.); 6150708006@vip.jiangnan.edu.cn (J.N.)

**Keywords:** LLTO, electrospinning, nanofiber, separator

## Abstract

A superfine Li_0.33_La_0.557_TiO_3_ (LLTO, 69.4 nm) was successfully synthesized by a facile solvent-thermal method to enhance the electrochemical properties of the lithium-ion battery separator. Co-axial nanofiber of cellulose and Poly(vinylidene fluoride-*co*-hexafluoropropylene) (PVDF-HFP) was prepared by a co-axial electrospinning technique, in which the shell material was PVDF-HFP and the core was cellulose. LLTO superfine nanoparticles were incorporated into the shell of the PVDF-HFP. The core–shell composite nanofibrous membrane showed good wettability (16.5°, contact angle), high porosity (69.77%), and super electrolyte compatibility (497%, electrolyte uptake). It had a higher ionic conductivity (13.897 mS·cm^−1^) than those of pure polymer fibrous membrane and commercial separator. In addition, the rate capability (155.56 mAh·g^−1^) was also superior to the compared separator. These excellent performances endowed LLTO composite nanofibrous membrane as a promising separator for high-performance lithium-ion batteries.

## 1. Introduction

High energy demands have accelerated emergent efforts to develop high-performance lithium-ion batteries (LIBs) due to their high specific energy and long cycle lifetime [1,2,3]. A separator in LIB is considered as a key component to prevent safety issues, because it can isolate the cathode and anode to prevent electrical short circuits and at the same time allow rapid transport of ionic charge carriers [4,5,6]. Polyolefin separators such as polyethylene (PE) and polypropylene (PP) have been widely used in LIBs [7]. However, their shrinkage at high working temperature leads to poor thermal property, which would cause a short circuit between electrodes [8]. Furthermore, the large difference in polarity between the polyolefin and electrolyte leads to high resistance and low electrolyte retention, which will influence the cycling performance of LIBs. To overcome these problems, some new materials and functional methods have been attempted for application in separators.

Semi-crystalline polyvinylidene fluoride (PVDF) and its copolymer Poly(vinylidene fluoride-*co*-hexafluoropropylene) (PVDF-HFP) have received special attentions as promising host polymers for separators of LIBs due to higher polarity and ionic conductivity [9,10]. Electrospinning is reported as one of the efficient methods to improve the wettability performance of the fibrous membrane due to its low diameter and high porosity [11,12]. In our previous study, a cellulose/PVDF-HFP nanofibrous membrane was successfully prepared by co-axial electrospinning. The composite membrane demonstrated good mechanical property, superior flame retardancy, excellent thermal stability and good electrolyte wettability [13].

Inorganic ceramics always attract great interest since they may present several advantages, *i.e.*, high electrochemical stability window, high thermal stability, high mechanical resistance [14,15,16]. Li-conducting ceramics based on Li_3*x*_La_2/3−*x*_TiO_3_ (named hereafter LLTO) hold an important place among them because of their high ionic conductivity at room temperature, *i.e.*, σ = 10^−3^ S·cm^−1^ for *x* = 0.10, and low electronic conductivity, *i.e.*, σ = 10^−8^ S·cm^−1^ [17,18]. Inspired by its excellent conductivity in Li-ion battery applications, various methods, such as the solid-state reaction method, sol–gel method and solutions precipitation, are applied to synthesize perovskite-type lithium ion-conducting oxides [19,20]. However, it was considered that the LLTOs reported in literature might not be suitable for mingling in nanofibers because of their large diameters (larger than 100 nm). In this work, a superfine Li_0.33_La_0.557_TiO_3_ was facilely synthesized by the solvent thermal method, and then was added into the shell of nanofibers which were co-axially electrospun with cellulose acetate and PVDF-HFP. The electrochemical properties and cycling performance of the lithium-ion batteries were evaluated and compared to those of commercial separator.

## 2. Experimental Section

### 2.1. Materials

Poly(vinylidene fluoride-*co*-hexafluoropropylene) (PVDF-HFP) (molecular weight 50,000 g·mol^−1^) was purchased from Shanghai 3F New Materials Co., Ltd (Shanghai, China). Cellulose acetate, lithium nitrate, lanthanum nitrate, butyltitanate, dimethylacetamide (DMAc) and acetone were supplied by Sinopharm Chemical Reagent Co., Ltd (Shanghai, China) and used without further purification. The electrolyte was composed of 1 M LiPF6 dissolved in ethylene carbonate, dimethyl carbonate, and ethylene methyl carbonate (1:1:1, *v*/*v*/*v*).

### 2.2. Preparation of LLTO Nanoparticles and Co-Axial Electrospinning of LLTO Composite Nanofibrous Membrane

Li_0.33_La_0.557_TiO_3_ nanoparticles were synthesized by lithium nitrate, lanthanum nitrate and butyl titanate with the starting stoichiometry amount 0.33:0.55:1.00, using a solution thermal method. Citric acid as the complexing agent was mixed in an ethanol solution. Provide quantitative of lithium nitrate and butyl titanate was added in the solution with stirring at 80 °C for 30 min. Lanthanum nitrate solution was then added to the reaction system in drops, followed by stirring for 80 min at ambient temperature. This solution was then transferred into a 200 mL Teflon-lined stainless steel autoclave and heated at 180 °C for 10 h. After slowly cooling it to room temperature, the dried mixture was paralyzed at 350 °C for 4 h and then calcined at 900 °C for 2 h with the heating rate of 5 °C/min. The calcined product was ground in an agate mortar and then sieved through #325 mesh for further use.

The two polymer solutions were independently fed through concentrically configured needles with outer and inner diameters of 1.3 and 0.3 mm, respectively. The shell solution was prepared by dissolving PVDF-HFP in a mixture of DMAc and acetone (3/7, *w*/*w*) to 10 wt% containing different amounts of LLTO particles (0, 2, 5 and 8 wt%). The core solution was prepared by dissolving cellulose acetate into DMAc and acetone (2/1, *w*/*w*) to 15 wt%. Coaxial electrospinning was conducted according to our previous work [13]. The as-prepared electrospun nanofibers were then dried in a vacuum oven at 60 °C for 12 h. Hydrolysis of cellulose acetate/PVDF-HFP membrane was performed in 0.05 M LiOH aqueous solution at ambient temperature for 10 h to produce composite nanofibrous membrane. Then the obtained membrane was rinsed in distilled water and dried under vacuum at 60 °C for 12 h.

### 2.3. Characterizations and Electrochemical Evaluation

The morphologies of LLTO particles and nanofibers were examined using a field emission scanning electron microscope (FE-SEM, Hitachi, Tokyo, Japan) and transmission electron microscope (TEM, JEM-2100HR, JEOL, Tokyo, Japan). The structure of LLTO particles was investigated using wide-angle X-ray diffraction (WXRD, D8, Bruker, Karlsruhe, Germany).

The thermal stability was analyzed by using a thermogravimetric analyzer (TGA, Q500, TA Instruments, New Castle, DE, USA) under a flowing nitrogen atmosphere at a heating rate of 10 C·min^−1^ with the temperature range of 30–900 °C.

The wettability was examined by a sessile drop method and the porosity of membranes was measured by immersing the membrane in *n*-butanol for 1 h [13].

The testing cells had a typical coin-type construction using lithium foils as both counter electrode and reference electrode. The cells were assembled in an argon-filled glove box.

Charge-discharge tests were carried out at a current density from 0.2 C to 5.0 C in a range of 2.8–4.2 V *vs.* Li/Li^+^. All the tests were performed at 20 °C. The ionic conductivity and interfacial resistances of LLTO/PVDF-HFP/cellulose nanofibrous membrane were evaluated using electrochemical impedance spectroscopy (EIS) measurement in combination with an Electrochemical Workstation (CHENHUA, CHI710b, Shanghai, China). The electrochemical stability of the separators was measured by a linear sweep voltammograms (LSV) on a working electrode of stainless-steel and a counter electrode of lithium metal at the potential range between 2.5 V and 6.0 V under the scan rate of 1.0 mV·s^−1^ at 20 °C.

## 3. Results and Discussion

### 3.1. Structure of LLTO and Composite Nanofibrous Membrane

**Figure 1:**
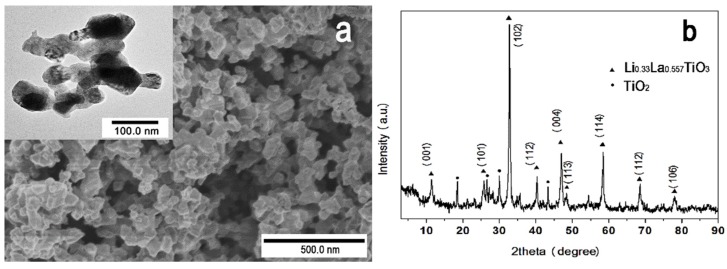
(**a**) SEM and TEM (inset) images and (**b**) XRD pattern of Li_0.33_La_0.557_TiO_3_ particles.

Figure 1 shows the uniformly nano-sized morphologies of the LLTO, after calcination at the temperature at which crystallization finishes (900 °C). It is observed from the images that the size of the particle ranges from 42 nm to 99 nm, with an average size of 69.4 nm, which is significantly finer than those of references listed in Table 1. The smaller diameter of the particles endows them as very suitable functional particles for doping in polymer nanofibers.

Figure 1b shows the powder X-ray diffraction pattern of LLTO particles sintered at 900 °C. The main phase is crystallized into an orthorhombic perovskite which depends on the preparation condition. The small amount of rutile TiO_2_ and unknown phases are observed from the XRD pattern, which might be produced during the sintering process. The average metal composition of the sintered sample is determined to be La:Li:Ti = 0.55:0.33:1 according to the standard atlas and references [22], which is consistent with the nominal composition.

**Table 1:** LLTO particles prepared by various methods.

Authors	Forms	Methods	Diameters
Abhilash *et al.* [21]	Li_0.5_La_0.5_TiO_3_	solid-state reaction	80 nm
Tang *et al.* [22]	Li_0.34_La_0.51_TiO_2.94_	solid-state reaction	100 nm
Hua *et al.* [23]	Li_0.27_La_0.54_TiO_2.945_	sol–gel	1.5 μm
Yoon *et al.* [24]	Li_0.35_La_0.55_TiO_3_	solid-state reaction	1.5–1.6 μm
Geng *et al.* [25]	Li_0.5_La_0.5_TiO_3_	sol–gel	200 nm
Murugesan *et al.* [26]	Li_0.3_La_0.566_TiO_3_	Pechini-type	100 nm
Liang *et al.* [19]	Li_0.125_La_0.625_TiO_3_	sol–gel	200 nm
Ionela *et al.* [27]	La_0.66_Li_0.33_TiO_3_	sol-gel	123.01 nm
Anatolii Belous *et al.* [28]	Li_0.5_La_0.5_TiO_3_	Solution precipitations	100 nm
Our work	Li_0.35_La_0.55_TiO_3_	Thermal-solution	69.4 nm

**Figure 2:**
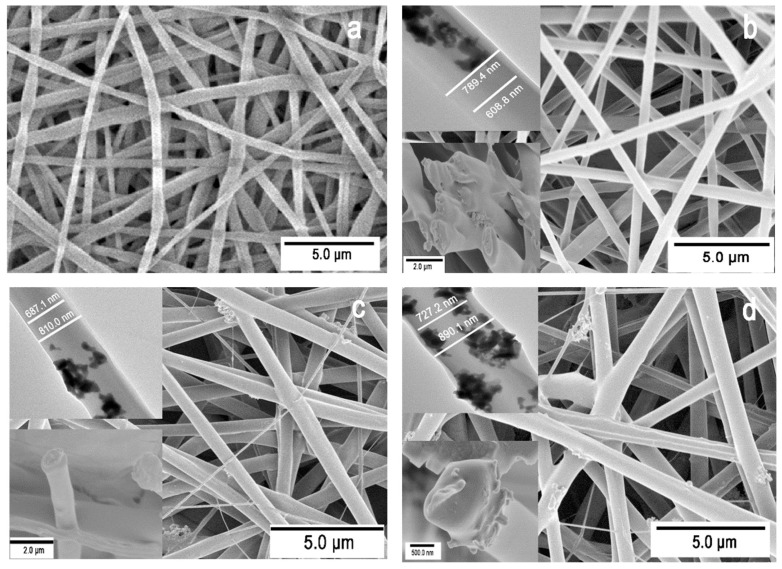
(**a**) SEM image of cellulose/PVDF-HFP nanofibers; (**b**) composite nanofibers containing 2% LLTO; (**c**) 5% LLTO and (**d**) 8% LLTO; inset images are TEM and SEM images of cross-sectional.

The morphological features of the composite nanofibrous membranes observed in SEM and TEM are shown in Figure 2. The diameter of core–shell nanofibers fabricated with a 3:3 flow rate ratio ranges from 652 nm to 779 nm, averaging at 712 ± 65 nm (Figure 2a). The average diameters of composite nanofibers containing 2%, 5% and 8% LLTO nanoparticles are 795 ± 89 nm (Figure 2b), 822 ± 76 nm (Figure 2c), and 888 ± 112 nm (Figure 2d), respectively, showing a little variation among all four samples. The cross-sectional structures of the three composite membranes show clear core–shell structures with apparently different core and shell thicknesses (inset SEM a images in Figure 2b–d), consistent with the biphasic fibers with a darker cellulose core and lighter PVDF-HFP shell in the TEM images (inset TEM images in Figure 2b–d). The addition of 2% LLTO leads to the formation of a small number of irregularities and uneven surface morphology, and the resultant LLTO composite nanofibers display a wider fiber diameter distribution. With further increase in LLTO content, the surface roughness of the composite nanofiber increases, and some LLTO particles begin to agglomerate and form clusters.

### 3.2. Thermal Stability

**Figure 3:**
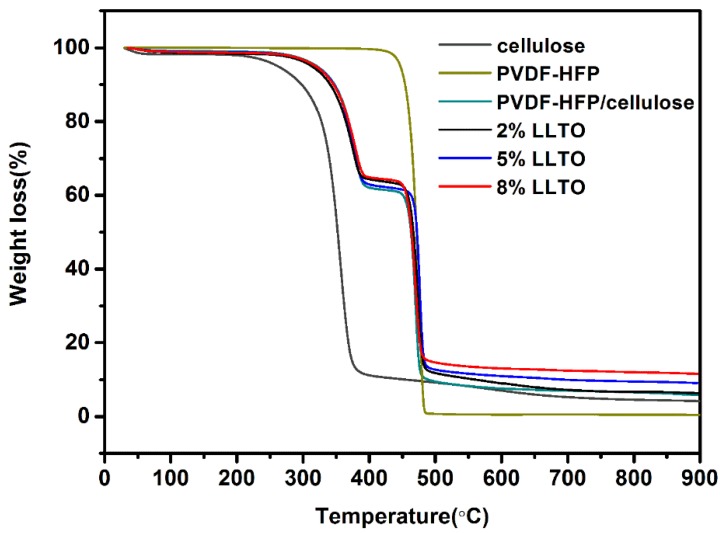
TGA curves of PVDF-HFP/cellulose and LLTO composite nanofibrous membrane.

The evaluation of the thermal properties of the composite nanofibrous membranes is very important for the determination of the performance of the separator in the range of temperatures in which the battery must be stable. Figure 3 shows the TGA results of the composite nanofibrous membranes. The LLTO composite nanofibrous membranes experience thermal degradation all around 300 °C (the temperature at which the weight loss of the sample is 1%), suggesting the excellent thermal properties originate from the intrinsic thermal stability of pure cellulose nanofibers (at 300 °C the weight loss of the sample is 10.5%). The separator containing 8% LLTO has the maximum decomposing temperature of 477 °C, and 11.8% of residues at 900 °C, which are slightly higher than 5% and 2% LLTO composite separator having 9.07% and 6.18% of residues, respectively. The enhanced decomposing temperature and reduced weight loss may be ascribed to the doping of LLTO nanoparticles and its efficiency to retard the thermal degradation of cellulose.

### 3.3. Liquid Electrolyte Uptake

**Figure 4:**
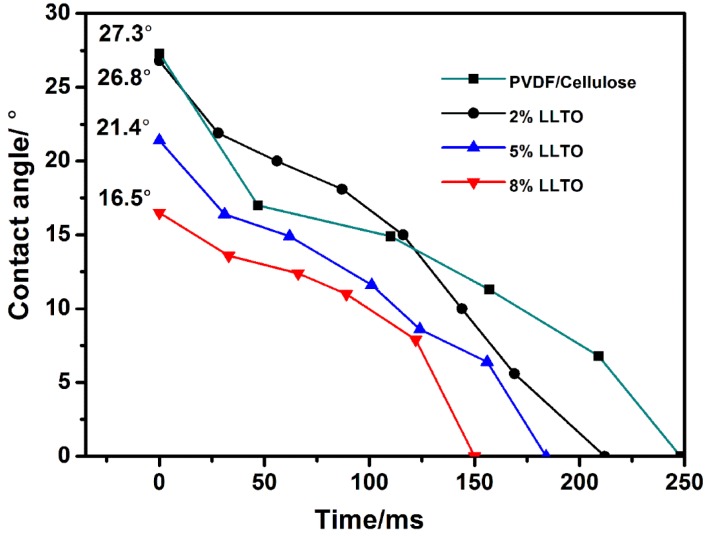
Surface contact angle of PVDF-HFP/cellulose and LLTO composite nanofibrous membrane.

A separator with good wettability can retain the electrolyte effectively and facilitate electrolyte diffusion smoothly. The wettability of the LLTO composite nanofibrous membranes was evaluated by contact angle measurements. Our previous study showed that the contact angle of the commercial separator was 69.29° [13], indicating its poor wettability. For the cellulose/PVDF-HFP core-shell nanofibrous membrane, the initiating contact angle is 27.3°, and the droplet of the electrolyte is instantly (2.1 s, Figure 4) infiltrated into the membrane. Initiating contact angles of membranes with LLTO doping is finally decreased to 16.5° (8% LLTO) due to the hydrophilic nature of LLTO, suggesting the better interfacial compatibility between the separator and electrolyte. Separator porosity is another critical factor for electrolyte uptake. The porosity and electrolyte uptake of composite nanofibrous membranes are shown in Table 2. The porosity of the nanofibrous membrane is fairly higher than that of the Celgard 2300 separator (47.88%). Both higher interfacial compatibility and porosity of the LLTO composite nanofibrous membrane lead to the higher electrolyte uptake of 497% (8% LLTO), *ca.* 255% and 40% higher than those of the Celgard 2300 separator (140%) and PVDF-HFP/cellulose nanofibrous membrane (355%).

**Table 2:** Porosity, electrolyte uptake, ionic conductivity of PVDF-HFP/cellulose and LLTO composite nanofibrous separators.

Separator	Thickness (μm)	Porosity (%)	Electrolyte Uptake (%)	Resistance (Ω)	Ionic Conductivity (mS·cm^−1^)
PVDF-HFP/cellulose	59	47.88	140	1.107	2.082
2% LLTO	48	66.36	355	0.415	4.518
5% LLTO	47	69.77	384	0.258	7.116
8% LLTO	37	67.45	487	0.104	13.897

### 3.4. Electrochemical and Battery Performance

**Figure 5:**
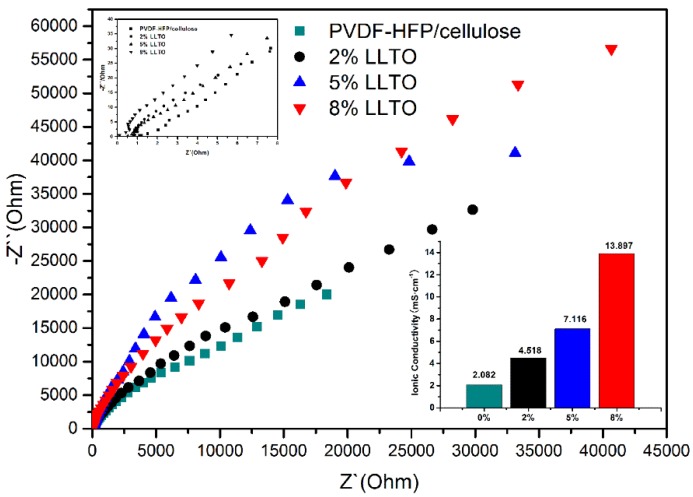
Nyquist plots of stainless steel/electrolyte-soaked separator/stainless steel cells at 20 °C, insets are magnified Nyquist plot and ionic conductivity of separators.

The ionic conductivity is the most important factor that directly affects the electrochemical performance of an electrolyte. Figure 5 shows the ionic conductivity dependence of the PVDF-HFP/cellulose membrane and its composites, the values of which are calculated from the Nyquist plots by intercepts of the plots shown in the inset of Figure 4. The ionic conductivity of the cellulose/PVDF-HFP nanofibrous separator is calculated to be 2.082 mS·cm^−1^, while the value of the commercial separator (Celgard^®^ 2300, Celgard, NC, USA) is 0.88 mS·cm^−1^ [13]. The value of the as-prepared composite membrane containing 8% LLTO is up to 13.897 mS·cm^−1^, which is much higher than that of the separator containing 2% LLTO (4.518 mS·cm^−1^) and 5% LLTO (7.116 mS·cm^−1^). The specifications and conductivity values of as-prepared separators are listed in Table 2. The higher ionic conductivity of the composite membranes might be attributed to the higher electrolyte uptake and the conductivity nature of LLTO particles.

**Figure 6:**
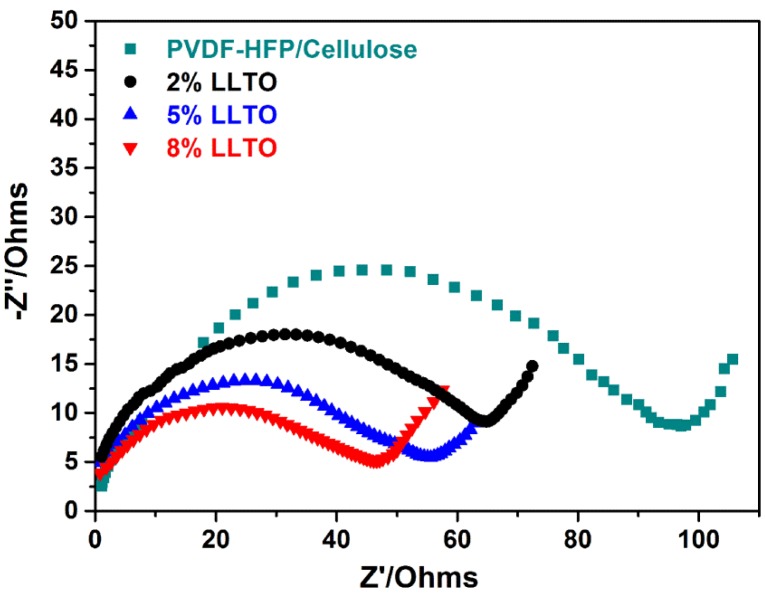
AC impedance spectra of Li/electrolyte-soaked separator/Li cells at 20 °C.

The interfacial properties of the separator with the Li electrode are another important aspect that affects the electrochemical performance of cell. Figure 6 shows the AC impedance spectra of the PVDF-HFP/cellulose membrane and its composites, the diameters of semi-circles represent the interfacial resistances between the Li electrode and the electrolyte-soaked separator. It can be seen from Figure 6 that interfacial resistances are 45, 55, 65 Ω for the composite membrane containing 8%, 5%, 2% LLTO, which are much higher than that of the PVDF-HFP/cellulose membrane (95 Ω). The smaller interfacial resistance means better compatibility between the separator and Li electrode.

**Figure 7:**
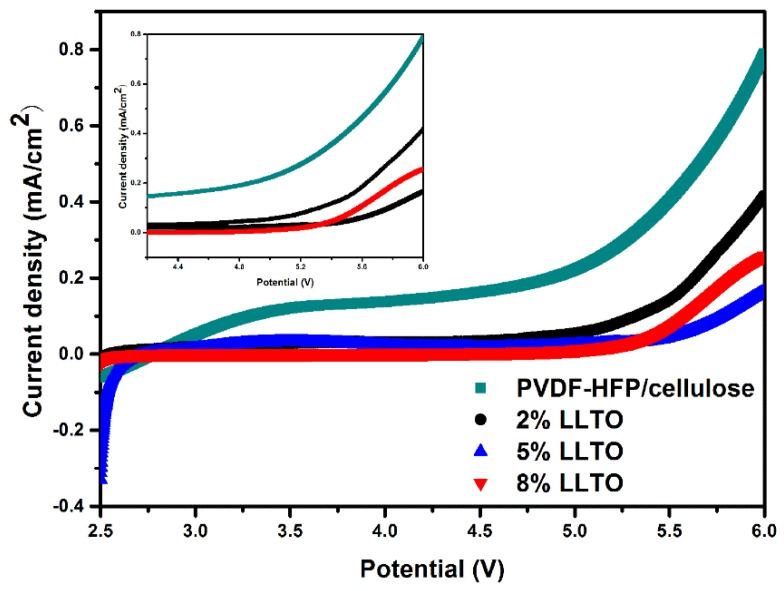
LSV curve of PVDF-HFP/cellulose and LLTO composite separators at a scan rate of 1.0 mV·s^−1^.

The electrochemical stability of the LLTO composite separator is conducted by linear sweep voltammograms testing to verify whether the composite separator is still stable in the electrochemical stability window. The results are shown in Figure 7 and it is clearly revealed that no appreciable decomposition of cellulose/PVDF-HFP and its composite separators takes place below 5.0 V *vs.* Li/Li^+^, which is almost comparable to that of the Celgard^®^ 2300 separator [13]. This result demonstrates the good electrochemical stability of the PVDF-HFP/cellulose and LLTO composite nanofibrous separator, revealing that it could be used as a promising alternative to the commercial separator for high-voltage lithium-ion batteries.

**Figure 8:**
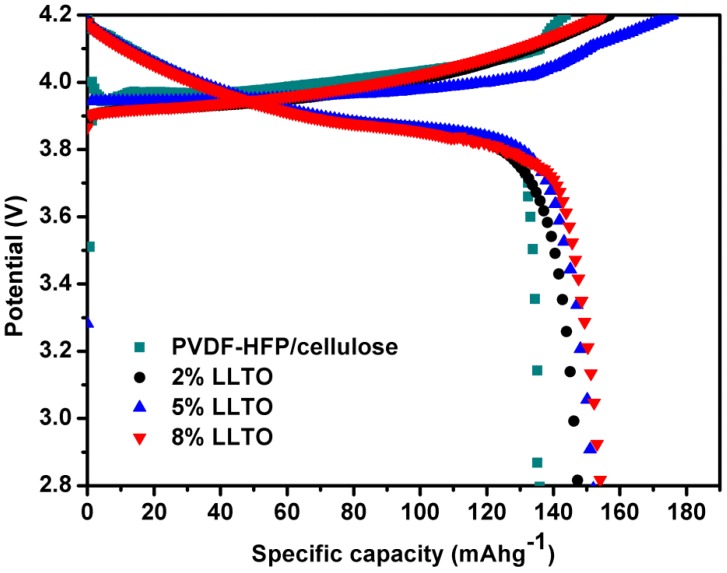
Charge-discharge characteristics of cells containing various separators at first cycle.

The electrochemical properties of PVDF-HFP/cellulose and its composite separators are investigated by charge and discharge testing and cycling performance characterization (Figure 8). The discharge capacity of batteries assembled with PVDF-HFP/cellulose is around 135.91 mAh·g^−1^ at 0.2 C, approximately 20% higher than the 114.8 mAh·g^−1^ discharge capacity for Celgard^®^ 2300 separators [13]. The capacities of the LLTO composite separator are found to be 148.00 mAh·g^−1^ (2% LLTO), 152.01 mAh·g^−1^ (5% LLTO) and 155.56 mAh·g^−1^ (8% LLTO) and are also superior to those in the references [13]. The utilization efficiency charge-discharge characteristics in the first cycle confirm that the composite membrane containing LLTO exhibits superior kinetic properties to those of commercial separators.

**Figure 9:**
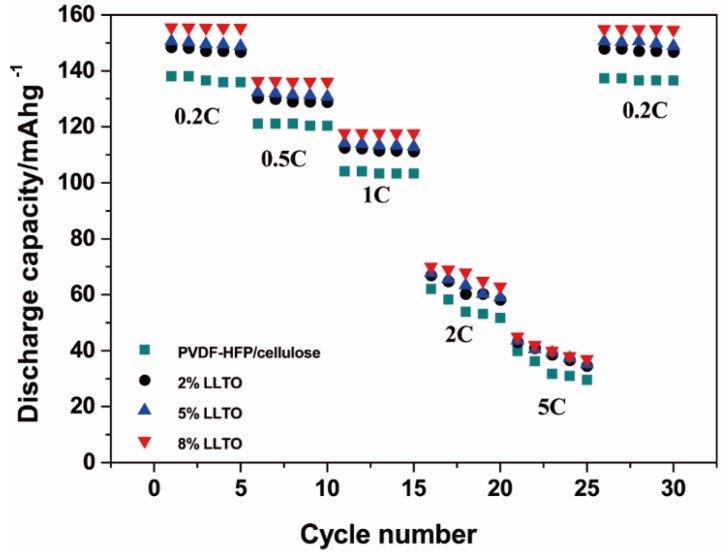
Rate performance of cells using PVDF-HFP/cellulose and LLTO composite nanofibrous membrane.

The cycle performances of the cells at different C-rates are shown in Figure 9. Similar rate capabilities are observed for those kinds of cells with various separators. With increased current densities of 0.2, 0.5, 1, 2 and 5 C, the discharge capacities decrease from 155.30 mAh·g^−1^ to 136.17, 117.65, 62.95 and 37.01 mAh·g^−1^ for cells containing 8% LLTO particles. The discharge capacity for the cell with composite separators fades with LLTO reducing from 8% to 2%. After being cycled at 5 C several times, the cycle rate is returned to 0.2 C. The discharge capacities are regained at 153.8 mAh·g^−1^ for the LLTO composite separator, which maintains about 98.9% of the initial discharge capacity. The superior discharge capacity and cycling performance could be ascribed to the higher ionic conductivity and better interfacial compatibility of the electrolyte-soaked composite nanofibrous membrane.

## 4. Conclusions

The superfine Li_0.33_La_0.557_TiO_3_ (LLTO, 69.4 nm) was successfully synthesized and characterized. It was found that the as-prepared membranes exhibited enhanced ionic conductivities due to the conductivity of LLTO particles. The composite LLTO nanofibrous membrane containing 8% LLTO was found to exhibit the highest ionic conductivity (13.897 mS·cm^−1^). Our LSV results also showed that the electrochemical stability of the composite nanofibrous membranes improved with an increase in the LLTO content. Moreover, cells assembled with the composite separator exhibited high storage and enhanced rate performances compared to the battery using the pure polymer nanofibrous separator. All these results suggest that this composite nanofiber membrane would be a promising separator for high-performance lithium-ion batteries.

## References

[1] Fang M.-D., Ho T.-S., Yen J.-P., Lin Y.-R., Hong J.-L., Wu S.-H., Jow J.-J. (2015). Preparation of advanced carbon anode materials from mesocarbon microbeads for use in high C-rate lithium ion batteries. Materials.

[2] Huang X.S. (2014). Evaluation of a polymethylpentene fiber mat formed directly on an anode as a battery separator. J. Membr. Sci..

[3] Liang X.X., Yang Y., Jin X., Huang Z.L., Kang F.Y. (2015). The high performances of SiO_2_/Al_2_O_3_-coated electrospun polyimide fibrous separator for lithium-ion battery. J. Membr. Sci..

[4] Shi C., Zhang P., Huang S.H., He X.Y., Yang P.T., Wu D.Z., Sun D.H., Zhao J.B. (2015). Functional separator consisted of polyimide nonwoven fabrics and polyethylene coating layer for lithium-ion batteries. J. Power Sour..

[5] Cai C., Wang Y. (2009). Novel nanocomposite materials for advanced Li-ion rechargeable batteries. Materials.

[6] Liu J.S., Li W.S., Zuo X.X., Liu S.Q., Li Z. (2013). Polyethylene-supported polyvinylidene fluoride–cellulose acetate butyrate blended polymer electrolyte for lithium ion battery. J. Power Sour..

[7] Xu Q., Kong Q.S., Liu Z.H., Wang X.J., Liu R.Z., Zhang J.J., Yue L.P., Duan Y.L., Cui G.L. (2014). Cellulose/Polysulfonamide Composite Membrane as a High Performance Lithium-Ion Battery Separator. ACS Sustain. Chem. Eng..

[8] Xiao S.Y., Yang Y.Q., Li M.X., Wang F.X., Chang Z., Wu Y.P., Liu X. (2014). A composite membrane based on a biocompatible cellulose as a host of gel polymer electrolyte for lithium ion batteries. J. Power Sour..

[9] Göeren A., Costa C.M., Tamaño Machiavello M.N., Cíntora-Juárez D., Nunes-Pereira J., Tirado J.L., Silva M.M., Gomez Ribelles J.L., Lanceros-Méndez S. (2015). Effect of the degree of porosity on the performance of poly (vinylidene fluoride-trifluoroethylene)/poly (ethylene oxide) blend membranes for lithium-ion battery separators. Solid State Ion..

[10] Seidel S.M., Jeschke S., Vettikuzha P., Wiemhoefer H.D. (2015). PVDF-HFP/ether-modified polysiloxane membranes obtained via airbrush spraying as active separators for application in lithium ion batteries. Chem. Commun..

[11] Shayapat J., Chung O.H., Park J.S. (2015). Electrospun polyimide-composite separator for lithium-ion batteries. Electrochim. Acta.

[12] Yanilmaz M., Zhang X.W. (2015). Polymethylmethacrylate/Polyacrylonitrile Membranes via Centrifugal Spinning as Separator in Li-Ion Batteries. Polymers.

[13] Huang F.L., Xu Y.F., Peng B., Su Y.F., Jiang F., Hsieh Y.L., Wei Q.F. (2015). Coaxial Electrospun Cellulose-Core Fluoropolymer-Shell Fibrous Membrane from Recycled Cigarette Filter as Separator for High Performance Lithium-Ion Battery. ACS Sustain. Chem. Eng..

[14] Zhu X.M., Jiang X.Y., Ai X.P., Yang H.X., Cao Y.L. (2015). A Highly Thermostable Ceramic-Grafted Microporous Polyethylene Separator for Safer Lithium-Ion Batteries. ACS Appl. Mater. Inter..

[15] Xu W.X., Wang Z.Y., Shi L.Y., Ma Y., Yuan S., Sun L.N., Zhao Y., Zhang M.H., Zhu J.F. (2015). Layer-by-Layer Deposition of Organic-Inorganic Hybrid Multilayer on Microporous Polyethylene Separator to Enhance the Electrochemical Performance of Lithium-Ion Battery. ACS Appl. Mater. Inter..

[16] Liang F., Hayashi K. (2015). A High-Energy-Density Mixed-Aprotic-Aqueous Sodium-Air Cell with a Ceramic Separator and a Porous Carbon Electrode. J. Electrochem. Soc..

[17] Inaguma Y., Nakashima M. (2013). A rechargeable lithium-air battery using a lithium ion-conducting lanthanum lithium titanate ceramics as an electrolyte separator. J. Power Sour..

[18] Piana M., Wandt J., Meini S., Buchberger I., Tsiouvaras N., Gasteiger H.A. (2014). Stability of a Pyrrolidinium-Based Ionic Liquid in Li-O_2_ Cells. J. Electrochem. Soc..

[19] Liang Y.Z., Ji L.W., Guo B.K., Lin Z., Yao Y.F., Li Y., Alcoutlabi M., Qiu Y.P., Zhang X.W. (2011). Preparation and electrochemical characterization of ionic-conducting lithium lanthanum titanate oxide/polyacrylonitrile submicron composite fiber-based lithium-ion battery separators. J. Power Sour..

[20] Zhang X.W., Ji L.W., Toprakci O., Liang Y.Z., Alcoutlabi M. (2011). Electrospun Nanofiber-Based Anodes, Cathodes, and Separators for Advanced Lithium-Ion Batteries. Polym. Rev..

[21] Abhilasha K.P., Christopher Selvina P., Nalinib B., Nithyadharsenic P., Pillai B.C. (2013). Investigations on pure and Ag doped lithium lanthanum titanate (LLTO) nanocrystalline ceramic electrolytes for rechargeable lithium-ion batteries. Ceram. Int..

[22] Tang H., Xu J. (2013). Enhanced electrochemical performance of LiFePO_4_ coated with Li_0.34_La_0.51_TiO_2.94_ by rheological phase reaction method. Mater. Sci. Eng. B.

[23] Hua C.X., Fang X.P., Wang Z.X., Chen L.Q. (2013). Lithium storage in perovskite lithium lanthanum titanate. Electrochem. Commun..

[24] Yoon J., Hunter G., Akbar S., Dutta P.K. (2013). Interface reaction and its effect on the performance of a CO_2_ gas sensor based on Li_0.35_La_0.55_TiO_3_ electrolyte and Li_2_CO_3_ sensing electrode. Sens. Actuators B Chem..

[25] Geng H.X., Lan J.L., Mei A., Lin Y.H., Nan C.W. (2011). Effect of sintering temperature on microstructure and transport properties of Li_3*x*_La_2/3−*x*_TiO_3_ with different lithium contents. Electrochim. Acta.

[26] Vijayakumar M., Inaguma Y., Mashiko W., Crosnier-Lopez M.P., Bohnke C. (2004). Synthesis of Fine Powders of Li_3*x*_ La_2/3-*x*_ TiO_3_ Perovskite by a Polymerizable Precursor Method. Chem. Mater..

[27] Popovici I.C., Chirila E., Popescu V., Ciupina V., Pordan G. (2007). Sol–gel preparation and characterization of perovskite lanthanum lithium titanate. J. Mater. Sci..

[28] Belous A., Yanchevskiy O., V’yunov O., Bohnke O., Bohnke C., Berre F.L., Fourquet J.L. (2004). Peculiarities of Li_0.5_La_0.5_TiO_3_ Formation During the Synthesis by Solid-State Reaction or Precipitation from Solutions. Chem. Mater..

